# Closing the circle

**DOI:** 10.7554/eLife.42507

**Published:** 2018-11-14

**Authors:** Marylou C Machingura, James V Moroney

**Affiliations:** 1 Department of BiologyGeorgia Southern UniversitySavannahUnited States; 2 Department of Biological SciencesLouisiana State UniversityBaton RougeUnited States

**Keywords:** Chlamydomonas reinhardtii, carbon fixation, microcompartment, pyrenoid, photosynthesis, rubisco, Other

## Abstract

In Chlamydomonas the different stages of the Calvin-Benson cycle take place in separate locations within the chloroplast.

**Related research article** Küken A, Sommer F, Yaneva-Roder L, Mackinder LCM, Höhne M, Geimer S, Jonikas MC, Schroda M, Stitt M, Nikoloski Z, Mettler-Altmann T. 2018. Effects of microcompartmentation on flux distribution and metabolic pools in *Chlamydomonas reinhardtii* chloroplasts. *eLife* **7**:e37960. doi: 10.7554/eLife.37960

When the unicellular green alga *Chlamydomonas reinhardtii* is viewed under a microscope, the most prominent object in the cell is a structure called the pyrenoid. Located within the chloroplast, the organelle where photosynthesis takes place, the pyrenoid was once thought to be a storage body composed of protein and starch. It subsequently became clear that, instead, the structure hosted most of the cell’s Rubisco, an enzyme that is responsible for the first step in photosynthesis (Rawat et al., 1996), and that the pyrenoid was in fact the main location for the fixation of CO_2_ in the cell.

Rubisco needs a high concentration of CO_2_ to work efficiently because it evolved when the Earth’s atmosphere contained almost 100 times as much CO_2_ as it does now: if the CO_2_ concentration gets low, a competing reaction with oxygen takes place, which significantly inhibits photosynthesis. Most unicellular algae have adapted to these lower levels of CO_2_ by developing a carbon concentration mechanism, a process that requires the Rubisco in Chlamydomonas to be packaged in the pyrenoid. Green algae are in the same lineage as terrestrial plants and both groups have Rubisco enzymes with similar properties. However, land plants rarely have pyrenoids because CO_2_ diffuses 10,000 times more rapidly in air than in water. Once an aquatic organism has fixed CO_2_, it takes a long time for the next molecule to arrive, hence the need for a carbon concentration mechanism that kicks in when the levels of CO_2_ get too low.

The carbon concentration mechanism in Chlamydomonas takes bicarbonate (HCO_3_^-^) from outside the cells and converts it into CO_2_ that can be used by Rubisco inside the pyrenoid. There, Rubisco catalyzes the addition of CO_2_ onto a molecule known as RuBP to create two molecules of a compound called 3PGA; this is the first step in the Calvin-Benson cycle, a chain of reactions which results in the creation of organic molecules that the cell needs. However, while it is established that Calvin-Benson cycle enzymes are inside the chloroplast, their exact location has been unclear in Chlamydomonas. Now, in eLife, Tabea Mettler-Altmann and colleagues – including Anika Küken as first author – report results that help to shed light on this mystery (Küken et al., 2018).

Küken et al. used multiple experimental approaches to identify where the other enzymes involved in the Calvin-Benson cycle were located. Cell cultures grown in low and high concentrations of CO_2,_ and hence with or without an active carbon concentration mechanism, were analyzed for Calvin-Benson cycle enzyme activities in isolated pyrenoids (following a method developed by Mackinder et al., 2016) and in whole cells. Amongst the Calvin-Benson enzymes, only Rubisco was largely detectable in the pyrenoid, with very small amounts in the stroma, the fluid that fills the inner cavity of the chloroplast. Since the activities of the other enzymes of the Calvin-Benson cycle were not in the pyrenoid, this indicated that they were in the stroma. The localization of each of these enzymes was further confirmed by fusing the proteins with a fluorescent ‘Venus’ reporter, and then using confocal microscopy to image the system. This imaging indicated that all of the Calvin-Benson cycle enzymes were in the stroma except Rubisco. The results suggested that a large number of 3PGA molecules moved from the pyrenoid to the stroma, and that there was also a large flux of RuBP from the stroma to the pyrenoid (Figure 1).

**Figure 1.**
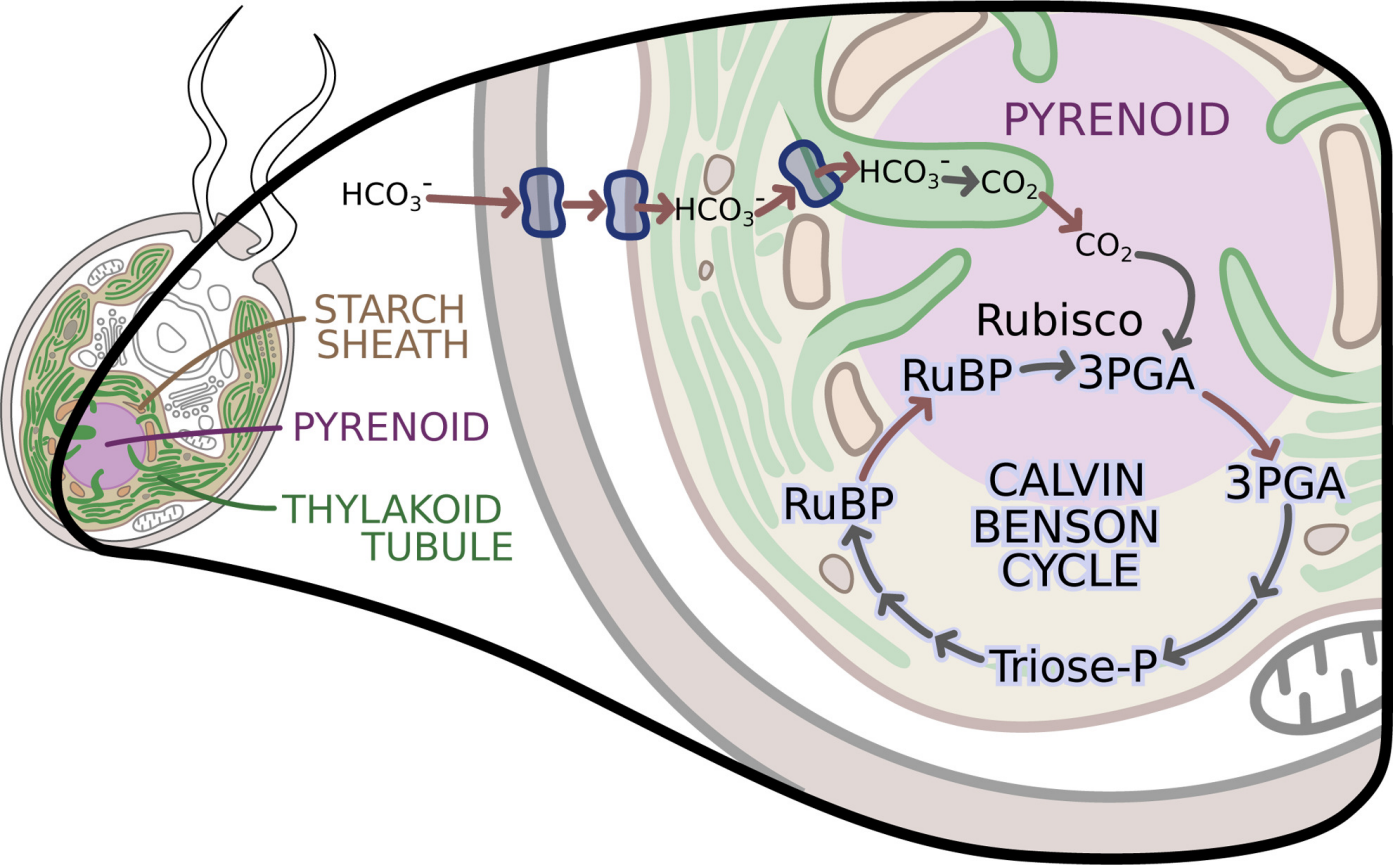
The enzymes of the Calvin-Benson cycle are in two locations. In Chlamydomonas, the Calvin-Benson cycle, which fixes CO_2_ and creates molecules that will become sugars, takes place in the cavity of the chloroplast or stroma (insert; pale yellow), and in the pyrenoid (purple), a sub-structure surrounded by a starch sheath (brown). The cycle involves the conversion of RuBP into 3PGA, which is then transformed into Triose-P, a compound that is then converted back into RuBP. The creation of 3PGA from RuBP, which takes place in the pyrenoid, is catalyzed by Rubisco and requires a supply of CO_2_. RuBP diffuses passively from the stroma into the pyrenoid, while 3PGA flows from the pyrenoid into the stroma, where the rest of the Calvin-Benson reactions take place. Enzyme-catalyzed reactions are shown with grey arrows, while movement of molecules, such as the diffusion of RuBP and 3PGA, is indicated with red arrows. A carbon concentration mechanism (top of figure) relies on transport proteins on the cell and chloroplast membranes that pump bicarbonate (HCO_3_^-^), a precursor for CO_2_, into the thylakoid tubules (Wang et al., 2015). There, enzymes called carbonic anhydrases convert the bicarbonate into CO_2_ (Aspatwar et al., 2018) before it makes its way into the pyrenoid. Image credit: Erin I Walsh (CC-BY 4.0)

Küken et al. – who are based at the Max Planck Institute of Molecular Plant Physiology, the University of Potsdam, the Carnegie Institution for Science, the University of Bayreuth and Heinrich Heine University – also quantified how much RuBP was bound to Rubisco and how much was free. They found that most of the bound RuBP was in the pyrenoid, with less free RuBP measured in the stroma. The concentration gradient of RuBP between the two compartments supports the idea that this metabolite moves by diffusion. Cells with an active carbon concentration mechanism, which have a higher percentage of Rubisco in the pyrenoid, also show higher fluxes of RuBP into the structure, and higher fluxes of 3PGA out of it.

The work of Küken et al. reinforces the description of the pyrenoid as a dynamic liquid matrix (Freeman Rosenzweig et al., 2017), surrounded by a starch sheath that does not seem to hinder the diffusion of RuBP and 3PGA between the pyrenoid and the stroma. Moreover, the stroma contains structures called thylakoid tubules, which also take part in photosynthesis. These tubules penetrate through the starch sheath (Figure 1; Engel et al., 2015), possibly increasing the surface area for the diffusion of the metabolites. The model proposed by Küken et al., with the Calvin-Benson cycle taking place in both the stroma and the pyrenoid, and Rubisco acting in the pyrenoid, supports this idea.

The pyrenoid is found in almost all aquatic photosynthetic organisms, although its morphology is different in diatoms, dinoflagellates and green algae (Bedoshvili et al., 2009; Heureux et al., 2017; Jeong et al., 2014). It will be interesting to see if Rubisco is always localized away from the rest of the Calvin-Benson cycle or whether there will be variations between these algal types.
